# Impacto de los programas de salud ERA y GES en la mortalidad por neumonía adquirida en la comunidad en personas de 65 años o más en Chile

**DOI:** 10.26633/RPSP.2019.41

**Published:** 2019-05-03

**Authors:** José Tomás Valdés, Claudia Contreras, Marcela Cárcamo, Pamela San Martín, Nicolás Valdés, Alyssa Sbarra, María Teresa Valenzuela

**Affiliations:** 1 Universidad de los Andes Universidad de los Andes Santiago Chile Universidad de los Andes, Santiago, Chile.; 2 Universidad de los Andes Universidad de los Andes Departamento de Salud Pública y Epidemiología Santiago Chile Departamento de Salud Pública y Epidemiología, Universidad de los Andes, Santiago, Chile.; 3 Universidad de los Andes Universidad de los Andes Vicedecanato de Investigación y Postgrado Santiago Chile Vicedecanato de Investigación y Postgrado, Universidad de los Andes, Santiago, Chile.; 4 Yale School of Public Health Yale School of Public Health Department of Epidemiology of Microbial Diseases New HavenConnecticut Estados Unidos de América Department of Epidemiology of Microbial Diseases, Yale School of Public Health, New Haven, Connecticut, Estados Unidos de América.

**Keywords:** Neumonía, anciano, anciano de 80 años o más, atención primaria de salud, Chile, Pneumonia, aged, aged, 80 and over, primary health care, Chile, Pneumonia, idoso, idoso de 80 anos ou mais, atenção primária à saúde, Chile

## Abstract

**Objetivo.:**

Evaluar el impacto del Programa de Enfermedades Respiratorias del Adulto (ERA) y el Régimen General de Garantías Explícitas en Salud (GES) en la mortalidad por neumonía adquirida en la comunidad (NAC) en personas de 65 años o más en Chile.

**Métodos.:**

En este estudio ecológico se calcularon las tasas anuales y trimestrales de mortalidad por NAC en personas de 65 a 79 años y de 80 años o más entre 1990 y 2014. Las fuentes de información fueron las bases de datos del Departamento de Estadística e Información de Salud y del Instituto Nacional de Estadística de Chile. Como intervenciones se evaluó el Programa ERA (puesto en marcha en el 2001) y la inclusión de la NAC en el GES (a partir del 2005). Los datos se analizaron mediante el método de series de tiempo interrumpidas, según el modelo de Prais-Winsten. Se consideró un nivel de significación del 5%.

**Resultados.:**

El análisis mostró que después del inicio del programa ERA se observaron disminuciones significativas de la tasa de mortalidad por NAC en los dos grupos de edad estudiados, mientras que a partir de la incorporación de la NAC al programa GES no se encontraron cambios estadísticamente significativos en esas tasas.

**Conclusiones.:**

La implementación del programa ERA contribuyó a reducir las tasas de mortalidad por NAC en personas de 65 años o más en Chile, no así la incorporación de la NAC al GES.

## Abstract (en)

**Objective.:**

Evaluate the impact of the Adult Respiratory Diseases (ERA) Program and the General System of Explicit Health Guarantees (GES) on mortality from community-acquired pneumonia (CAP) in persons aged ≥65 years in Chile.

**Methods.:**

In this ecological study, annual and quarterly mortality rates from CAP were calculated in persons aged 65 to 79 years and ≥80 years from 1990 to 2014. Information was gathered from the databases of Chile’s Department of Health Statistics and Information and its National Statistics Institute. The ERA Program (implemented in 2001) and the inclusion of CAP in the GES (starting in 2005) were evaluated as interventions. Data were analyzed using the interrupted time-series method, following the Prais-Winsten model, with a 5% significance level.

**Results.:**

The analysis showed that after the ERA Program began, significant reductions in CAP mortality were observed in the two age groups studied, whereas after CAP was added to the GES program, no statistically significant changes were found in those rates.

**Conclusions.:**

Implementation of the ERA Program helped to reduce CAP mortality in persons aged ≥65 years in Chile, whereas inclusion of CAP in the GES program did not.

## RESUMO

**Objetivo.:**

Avaliar o efeito do Programa Enfermidades Respiratorias del Adulto (doenças respiratórias do adulto, ERA) e do Régimen General de Garantías Explícitas en Salud (regime geral de garantias explícitas em Saúde, GES) na mortalidade por pneumonia adquirida na comunidade (PAC) em indivíduos acima de 65 anos no Chile.

**Métodos.:**

Estudo ecológico em que foram calculadas as taxas anuais e trimestrais de mortalidade por PAC em indivíduos de 65 a 79 anos e acima de 80 anos no período entre 1990 e 2014. Os dados foram obtidos dos bancos de dados do Departamento de Estatística e Informação em Saúde e do Instituto Nacional de Estatística do Chile. As intervenções avaliadas foram o Programa ERA (implantado em 2001) e a inclusão da PAC no GES (a partir de 2005). Os dados foram analisados com o método de séries temporais interrompidas segundo o modelo de Prais-Winsten. O nível de significância foi de 5%.

**Resultados.:**

A análise demonstrou que, após a implantação do Programa ERA, houve uma redução significativa na taxa de mortalidade por PAC nas duas faixas etárias consideradas. Por outro lado, a inclusão da PAC no GES não teve efeito estatisticamente significativo na taxa de mortalidade.

**Conclusões.:**

A implantação do Programa ERA contribuiu para reduzir a taxa de mortalidade por PAC em indivíduos acima de 65 anos no Chile, porém o mesmo não ocorreu com a inclusão da PAC no GES.

Las infecciones de las vías respiratorias bajas, como la neumonía adquirida en la comunidad (NAC), se ubican como la tercera causa de muerte en el mundo y la primera entre las enfermedades infecciosas; en el año 2015 causaron 3,2 millones de muertes. Aunque como causa de muerte en la Región de las Américas bajó a la sexta posición, se mantiene como la primera causa de muerte de origen infeccioso, con 318 000 fallecimientos registrados en 2015 (1). La NAC, que compromete el parénquima pulmonar, es una afección respiratoria aguda ocasionada por infecciones adquiridas fuera del ambiente hospitalario (2) por *Streptococcus pneumoniae*, *Mycoplasma pneumoniae*, *Haemophilus influenzae*, *Legionella pneumophila *y varios virus respiratorios como el de la influenza y la parainfluenza*,* entre otros (3). Los factores de riesgo identificados son el consumo de tabaco (4), la edad (5) y la coexistencia con inmunodeficiencias; el grupo de mayor riesgo está constituido por las personas de 65 años o más con enfermedades concomitantes (6).

Chile presenta un envejecimiento acelerado y constante, y las personas de 65 años o más representan el grupo poblacional con mayor crecimiento: 6,6%, 8,1% y 11,4% en los años 1992, 2002 y 2014, respectivamente (7). Sin embargo, a partir del año 2000 el país experimentó un descenso notable y sistemático de la mortalidad por NAC en personas de ese grupo de edad.

Entre los años 1997 y 2003, las enfermedades respiratorias ocuparon el tercer lugar entre las causas de muerte en Chile; de ellas, el 41% correspondió a las muertes por neumonía, especialmente en personas de 65 años o más, a las que correspondió el 90% de esas muertes (8). Para enfrentar esta problemática, en el 2001 se estableció el programa Enfermedades Respiratorias del Adulto (ERA) (9), implementado en los consultorios de atención primaria de salud (APS) y dirigido de manera priorizada a las personas de 65 años de edad o más; su objetivo general ha sido y es reducir la mortalidad y la letalidad de las enfermedades respiratorias del adulto en el país. En el marco de este programa se habilitaron salas de apoyo respiratorio del adulto (SARA) para facilitar el control y la atención de estos pacientes —de acuerdo con las normas establecidas para cada enfermedad— y, de esta manera, mejorar la capacidad resolutiva del nivel primario de atención. En el caso de la NAC, se establecieron indicaciones sobre la forma de hacer el diagnóstico, los criterios de hospitalización, los diagnósticos diferenciales, la categorización de los pacientes y las medidas para el tratamiento de la enfermedad (10).

En el año 2004 —y como parte de la reforma de salud en Chile— se promulgó la Ley 19.966 (11) que establece el programa denominado Plan de Acceso Universal a Garantías Explícitas en Salud (AUGE), luego renombrado Régimen General de Garantías Explícitas en Salud (GES) (12). Este programa se diseñó para facilitar el acceso a la salud de la población mediante un plan que garantizara a todos los beneficiarios el derecho a prestaciones mínimas. En esta canasta de prestaciones se incluyen productos y servicios, se especifican plazos de espera y se detallan protocolos de atención específicos para un grupo de enfermedades consideradas prioritarias, entre ellas la NAC (13). Las SARA siguen funcionando hasta el día de hoy y trabajan en conjunto con el GES para enfermedades respiratorias.

Así, el programa ERA está encargado de mejorar la capacidad de resolución de los servicios de APS con respecto al diagnóstico de la NAC (incluida la radiografía de tórax), mientras el GES debe garantizar el acceso oportuno a servicios de calidad y la protección financiera según el seguro de salud del asociado, todo esto ajustado a los tiempos máximos de espera para el otorgamiento de las prestaciones ofrecidas por entidades acreditadas o certificadas, según se establece en la Ley de la Prestación de Salud (14). En otras palabras, el programa debe garantizar la confirmación diagnóstica —clínica y radiológica— y el tratamiento farmacológico en las primeras 48 horas, además del tratamiento kinesiológico y otros procederes pertinentes.

Dentro de este marco de acción, el Ministerio de Salud de Chile confecciona las guías clínicas, que consisten en recomendaciones dirigidas a apoyar la toma de decisiones clínicas y priorizar algunas enfermedades, todo esto según se define en la ley (10). Es así como en el 2005 se publicó la primera de estas guías para la NAC en adultos de 65 años o más, actualizada en 2011 (15).

Tomando en cuenta las elevadas tasas de prevalencia y mortalidad de la NAC y las diversas iniciativas y esfuerzos puestos en marcha, en este trabajo se evalúa el impacto de los programas de salud ERA y GES en la mortalidad por NAC en personas de 65 años o más en Chile.

## MATERIALES Y MÉTODOS

Para este estudio ecológico de series de tiempo (16) se utilizaron datos secundarios de defunciones de 1990 a 2014 provistos por el Departamento de Estadísticas e Información de Salud (17), perteneciente al Ministerio de Salud de Chile. Para los datos de población se utilizaron las bases de datos del Instituto Nacional de Estadísticas de Chile (8).

Las causas de muertes tomadas en consideración corresponden, según la Clasificación Internacional de Enfermedades (CIE-9 y CIE-10) (18-21), a los códigos del 480 al 486 y del J12 al J18, respectivamente. Se utilizó la CIE-9 para las defunciones ocurridas entre los años 1990 y 1996 y la CIE-10 para las defunciones comprendidas entre los años 1997 y 2014, según dos grupos de edades: de 65 a 79 años y de 80 años o más, para ambos sexos.

A partir de estos datos se contabilizó el número total de muertes por NAC para cada año y trimestre.

### Análisis de los datos

Se calculó la tasa de mortalidad específica por NAC por 10 000 habitantes para cada año y trimestre en los dos grupos de edad. Como denominador se utilizó el número total de personas en cada grupo poblacional en cada año estudiado.

Para evaluar el impacto de la implementación del programa ERA (establecido en 2001) y la inclusión de la NAC en el GES (a partir del 2005), se utilizó el método de series de tiempo interrumpidas según el modelo de Prais-Winsten (22); se consideró como variable de respuesta el número de muertes por NAC, expresado como tasa de mortalidad por 10 000 habitantes.

Para poder aplicar este método, se consideró como etapa anterior a la puesta en marcha del programa ERA (pre-ERA) el período del 1990 al 2000, y se tomaron los años del 2002 al 2014 como período posterior al inicio de la intervención (pos-ERA); el año 2001 se consideró de transición.

Para evaluar el impacto del GES se consideró el período del 1990 al 2005 como etapa pre-GES, el período del 2007 al 2014 como etapa posterior al inicio de la intervención (pos-GES) y el 2006 como año de transición.

Mediante el análisis de series de tiempo interrumpidas se evaluó el impacto de las intervenciones (*Y*) en un período dado (*t*) a partir de las tasas de mortalidad por NAC, según la siguiente fórmula:

$$Y_{t}\text{ = }\beta_{0}+(\beta_{1}\cdot t)+(\beta_{2}\cdot g)+(\beta_{3}\cdot t\cdot g)$$

donde,

*t*: tiempo medido en años

*β*_0_: tasa de mortalidad inicial (*t* = 0)

*β*_1_: cambio en la tasa de mortalidad, según el tiempo

*β*_2_: cambio en el nivel de la tasa de mortalidad en el tiempo una vez que se aplicó la intervención

*β*_3_: cambio en la pendiente de la tasa de mortalidad en el tiempo una vez que se aplicó la intervención

*g*: variable auxiliar (*g* = 0 si las defunciones ocurrieron en la etapa previa a cada intervención y *g* = 1 si ocurrieron en la etapa posterior a cada intervención)

Los datos se colectaron en una hoja de cálculo de MS Excel. Se analizaron los resultados mediante estadística descriptiva (medianas, mínimos y máximos, frecuencias absolutas y relativas, según la variable analizada) por grupo de edad. Las variaciones porcentuales se calcularon en relación con las tasas de mortalidad por grupo de edad y programa de salud en cuestión. Para el análisis de series de tiempo interrumpidas se escogió un nivel de significación de 0,05 (5%). Los datos se procesaron mediante el paquete estadístico Stata versión 14.0.

Dado que el estudio es de carácter epidemiológico poblacional a partir de información pública recogida en bases de datos y los datos se trabajaron de forma anónima, no se requirió la aprobación del comité de ética.

## RESULTADOS

El número total de defunciones en la población estudiada fue de 93 870; de ellas, 64 336 (68,5%) eran personas de 80 años o más. La mediana de defunciones por NAC en el grupo de 65 a 79 años en los años estudiados (de 1990 a 2014) fue de 886 muertes anuales (mínimo: 572; máximo: 2 087). Mientras, la mediana de defunciones por NAC en el grupo de personas de 80 años o más fue de 2 425 muertes anuales (mínimo: 2 026; máximo: 3 792).

### Efecto del programa ERA (1990-2000 *vs.* 2002-2005)

En el grupo de 65 a 79 años, la tasa de mortalidad por NAC entre los años 1990 y 2000 (etapa pre-ERA) se redujo 56,5% (de 28,97 por 10 000 habitantes a 12,60 por 10 000 habitantes, respectivamente); en el período 2002-2005, la tasa se redujo 26,0% (de 8,63 por 10 000 habitantes a 6,39 por 10 000 habitantes, respectivamente) (cuadro 1).

Por su parte, la tasa de mortalidad registrada por NAC en el grupo de personas de 80 años o más en la etapa pre-ERA disminuyó 32,4% (de 175,29 por 10 000 habitantes a 118,53 por 10 000 habitantes, respectivamente); en la etapa pos-ERA, la tasa se redujo 9,8% (de 92,05 por 10 000 habitantes a 83,07 por 10 000 habitantes, respectivamente) (cuadro 1).

### Efecto de la inclusión de la NAC en el GES (1990-2005 *vs.* 2007-2014)

La tasa de mortalidad por NAC en el grupo de 65 a 79 años entre los años 1990 y 2005 (pre-GES) se redujo 77,9% (de 28,97 por 10 000 habitantes a 6,39 por 10 000 habitantes, respectivamente); en la etapa pos-GES, la tasa se redujo 7,0% (de 6,30 por 10 000 habitantes a 5,86 por 10 000 habitantes, respectivamente), con valores menores en los años 2011 y 2012 (4,51 y 5,74 por 10 000 habitantes, respectivamente) (cuadro 1).

**CUADRO 1:** Defunciones por neumonía adquirida en la comunidad por grupo de edad. Chile, 1990-2014

Programa ERA	Programa GES	Año	Población		Defunciones		Tasa, por 10 000 habitantes	
			65-79 años	80 años o más	65-79 años	80 años o más	65-79 años	80 años o más
Etapa pre-ERA	Etapa pre-GES	1990	662 387	138 346	1 919	2 425	28,97	175,29
		1991	685 248	143 695	1 523	2 123	22,23	147,74
		1992	708 109	149 044	1 537	2 350	21,71	157,67
		1993	730 969	154 393	1 740	2 627	23,80	170,15
		1994	753 830	159 743	1 658	2 659	21,99	166,45
		1995	776 690	165 091	2 051	3 343	26,41	202,49
		1996	801 506	172 620	2 087	3 358	26,04	194,53
		1997	826 322	180 149	1 816	3 250	21,98	180,41
		1998	851 139	187 679	1 651	3 354	19,40	178,71
		1999	875 955	195 207	1 890	3 792	21,58	194,26
		2000	900 771	202 736	1 135	2 403	12,60	118,53
Transición		2001	928 119	212 721	1 010	2 118	10,88	99,57
Etapa pos-ERA		2002	955 470	222 707	825	2 050	8,63	92,05
		2003	982 818	232 693	684	2 026	6,96	87,07
		2004	1 010 168	242 679	806	2 240	7,98	92,30
		2005	1 037 517	252 664	663	2 099	6,39	83,07
	Transición	2006	1 074 764	265 734	594	2 026	5,53	76,24
	Etapa pos-GES	2007	1 112 009	278 803	701	2 691	6,30	96,52
		2008	1 149 254	291 873	673	2 154	5,86	73,80
		2009	1 186 501	304 943	733	2 216	6,18	72,67
		2010	1 223 746	318 013	802	2 575	6,55	80,97
		2011	1 269 002	331 712	572	2 148	4,51	64,75
		2012	1 314 257	345 413	755	2 596	5,74	75,16
		2013	1 359 514	359 112	886	2 896	6,52	80,64
		2014	1 404 769	372 813	823	2 817	5,86	75,56

***Fuente:*** Elaboración propia.

Un comportamiento similar se observó en el grupo de personas de 80 años o más: entre los años 1990 y 2005 la tasa de mortalidad registrada por NAC disminuyó 52,6% (de 175,29 por 10 000 habitantes a 83,07 por 10 000 habitantes, respectivamente), mientras en la etapa pos-GES la tasa se redujo 21,7% (de 96,52 por 10 000 habitantes a 75,56 por 10 000 habitantes, respectivamente), con valores menores en los años 2009 y 2011 (72,67 y 64,75 por 10 000 habitantes, respectivamente) (cuadro 1).

**FIGURA 1:**
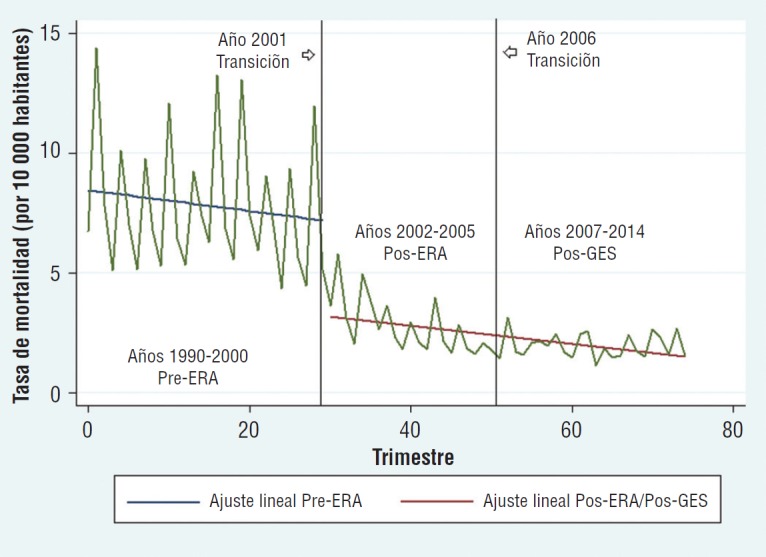
Tasa trimestral de mortalidad por neumonía adquirida en la comunidad en personas de 65 a 79 años, según el modelo de series de tiempo interrumpidas. Chile, 1990-2014

***Fuente:*** elaboración propia.

**Nota:** Transición: Se refiere al período de tiempo desde que se inicia una intervención hasta alcanzar una cobertura estable; ERA: Programa de Enfermedades Respiratorias del Adulto; GES: Régimen General de Garantías Explícitas en Salud.

**FIGURA 2:**
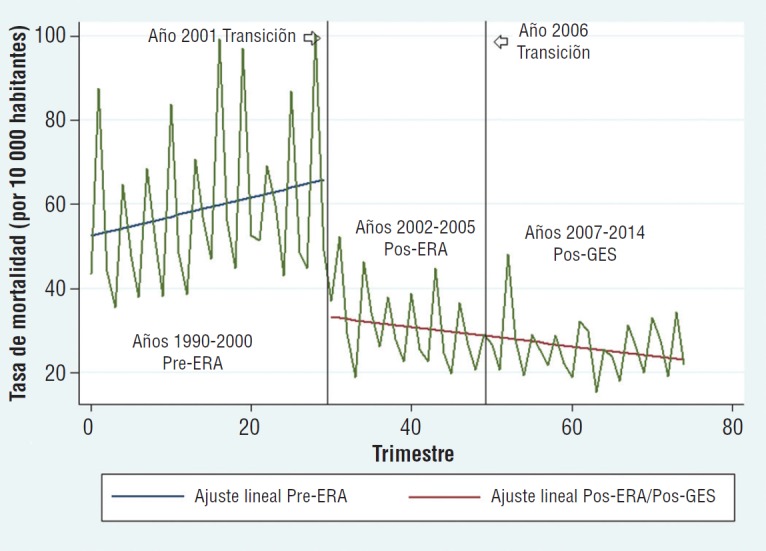
Tasa trimestral de mortalidad por neumonía adquirida en la comunidad en personas de 80 años o más de edad, según el modelo de series de tiempo interrumpidas. Chile, 1990-2014

***Fuente:*** elaboración propia.

**Nota:** Transición: Se refiere al período de tiempo desde que se inicia una intervención hasta alcanzar una cobertura estable; ERA: Programa de Enfermedades Respiratorias del Adulto; GES: Régimen General de Garantías Explícitas en Salud.

### Análisis de series de tiempo interrumpidas

Las tendencias de la mortalidad en el grupo de 65 a 79 años en las etapas anterior y posterior al inicio del programa ERA tuvieron comportamientos similares, sin embargo, durante la etapa pos-GES, las disminuciones fueron menores.

Al analizar las variaciones trimestrales, se encontró una situación similar y se advirtió, además, una disminución de la mortalidad en el período posterior a la implementación de los programas ERA y GES (figura 1).

Con relación a la población de 80 años o más, en la que ocurrió una disminución de las tasas de mortalidad después del año 2000, se observó una diferencia entre las tendencias de mortalidad previas y posteriores al programa ERA —ascendentes y posteriormente abruptamente descendentes—, fenómeno que se repite en las variaciones trimestrales (figura 2).

Después del inicio del programa ERA, con las SARA, se observaron cambios estadísticamente significativos en la mortalidad por NAC en el grupo de edad de 65 a 79 años (*p* < 0,001) y en el de 80 años o más (*p* < 0,05), mientras que el descenso de la mortalidad posterior a la implementación del GES, si bien es importante, no resultó significativo (*p* > 0,05).

## DISCUSIÓN

Como se observa en estos resultados, tras la implementación del programa ERA en Chile en el año 2001 (10) se observó una reducción en la mortalidad por NAC en los dos grupos poblacionales estudiados (personas de 65 a 79 años y de 80 años o más), con un descenso significativo de las tasas de defunción. Sin embargo, es importante destacar la disminución de la tasa de mortalidad en el grupo de 65 a 79 años de edad antes de la instauración del programa ERA (años 1990-2000), que puede deberse a la adopción de normas y recomendaciones nacionales propuestas por la Sociedad Estadounidense del Tórax (23) y la Sociedad Chilena de Enfermedades Respiratorias (24). Entre esas normas y recomendaciones se destaca la clasificación de los pacientes en grupos de riesgo según su edad, la gravedad de la enfermedad, las enfermedades concomitantes, la necesidad de hospitalización y el agente infeccioso involucrado, lo que contribuyó a establecer el pronóstico y la directriz terapéutica adecuada a cada grupo de edad.

La edad es un factor de riesgo independiente para la NAC y una mayor edad se ha asociado con una mayor mortalidad por esta enfermedad (25), por ello en el grupo de personas de 80 años o más las tasas de defunción son mayores que en el grupo de 65 a 79 años; esto puede deberse, al menos en parte, a la fisiopatología y las enfermedades concomitantes propias del adulto mayor (26). En este sentido, se debe considerar que en las personas de mayor edad, la NAC puede presentarse sin síntomas respiratorios o fiebre y manifestarse solo con síntomas inespecíficos, tales como decaimiento, anorexia o estado mental alterado, lo que puede retrasar el diagnóstico y afectar de forma negativa al pronóstico de estos pacientes (15). Además, el propio envejecimiento provoca alteraciones en la mecánica respiratoria con disminución de la fuerza y la tolerancia a la fatiga de los músculos respiratorios, algo que puede empeorar por otras enfermedades asociadas (25, 27).

Se observó también una disminución constante en las tasas de mortalidad, tanto antes como después de las intervenciones estudiadas, excepto en la población de 80 años o más durante la etapa pre-ERA (figura 2). Este declive puede deberse a factores exógenos no incluidos en este estudio (ver limitaciones), como una mayor inversión en la salud: el producto interno bruto (PIB) de Chile, que llegó a US$23 004 per cápita en el año 2016, dedicó US$1 915 (8,32%) per cápita a la salud (28-30), aunque no queda claro cuánto de ello se dirigió al diagnóstico y tratamiento de los casos de NAC. También puede haber influido que el nivel de escolaridad aumentó de 6 a 8 años en este mismo grupo y en el mismo período (31).

Al analizar estos resultados se deben tomar en cuenta algunas limitaciones. En primer lugar, el impacto de las intervenciones se midió a través de la mortalidad, lo que no incluye otras variables relevantes en el proceso enfermedad-muerte, tales como los factores biosociodemográficos —como el medio ambiente, el nivel socioeconómico de las personas, el nivel de acceso a la salud y las posibles redes de apoyo— que pudieran influir en el fenómeno estudiado. Además, se debe tomar en cuenta la introducción en el mercado de la vacuna antineumocócica PPSV23 en 1983. Esta vacuna polisacarídica, que abarca los 23 serotipos (32) considerados responsables de la mayor parte de las infecciones causadas por neumococos (33), se incluyó en el Programa Nacional de Inmunizaciones en el 2007, con la recomendación de aplicarse a todas las personas mayores de 75 años, algo que se extendió en el 2010 a todas las personas de 65 años o más (34). No obstante, si bien se ha demostrado la eficacia de esta vacuna en la prevención de enfermedades neumocócicas invasivas (35), no se ha determinado su capacidad de prevenir o disminuir la mortalidad por NAC. En Chile, la eficacia de la vacuna para prevenir la NAC tampoco se ha estudiado y las coberturas de vacunación alcanzadas son bajas (36).

No obstante estas limitaciones, los resultados de este estudio permiten concluir que la implementación del programa ERA contribuyó a reducir las tasas de mortalidad por NAC en personas de 65 años o más en Chile, no así la incorporación de la NAC al GES.

Junto a la incorporación de programas específicos para abordar problemas de salud pública relevantes como la NAC, se hace necesario recurrir a métodos actualizados para analizar su eficacia en el tiempo, con evaluaciones periódicas y continuas que permitan conocer su impacto en la población.

## Contribución de los autores.

Los autores JTV, CCB, PSM y MTV concibieron y planificaron el estudio; MC, PSM, NVO, ASB y MTV recolectaron los datos; todos los autores analizaron los datos; MC, PSM y MTV, interpretaron los resultados; JTV, CCB, MC, PSM y MTV escribieron el manuscrito. Todos los autores revisaron y aprobaron la versión final.

## Declaración.

Las opiniones expresadas en este manuscrito son responsabilidad de los autores y no reflejan necesariamente los criterios ni la política de la RPSP/ PAJPH y/o de la OPS.
